# Supplementary material for: Increase in the community circulation of ciprofloxacin-resistant Escherichia coli despite reduction in antibiotic prescriptions
Source: Commun Med (Lond). 2023 Aug 12;3:110. doi: 10.1038/s43856-023-00337-2 (PMC10421857; doi:10.1038/s43856-023-00337-2)
Supplement: Supplementary file 1 — Supplementary Information [file 43856_2023_337_MOESM1_ESM.pdf]

Supplementary material

Table of contents

Supplemental Methods

Supplemental Table S1. FQR *E. coli* clones identified in 2015 and 2021 studies

Supplemental Table S2. Virulence loci variably present in 158 FQREC from 151 fecal samples from 2015 (N=24) and 2021 (N=127) study collections

Supplemental Table S3. PMQR determinants identified in 2015 and 2021 FQR E. coli isolates

Supplemental Table S4. BLA loci identified in 129 3GCR FQREC isolates from 2015 and 2021

Supplemental Table S5. Cumulative antibiogram of FQREC

Supplemental Table S6. Types of QRDR mutations and their prevalence across major FQREC clonal groups

Supplemental Figure S1: Prescription rates of fluoroquinolones (FQ) and third and higher generation cephalosporins (including carbapenems, 3GC+) among Medicare enrollees in 2013-2020 in Washington state (closed circles) and USA (open circles).

Supplemental Data 1: List of FQREC from 2015 and 2021 fecal samples with the results of any testing performed on them (sequencing, antimicrobial resistance, PMQR,

BLA and VF determinants, number and type of QRDR mutations in GyrA and ParC and ciprofloxacin MIC.

Supplemental data 2: Sequences of gyrA alleles in fasta format**.**

Supplemental data 3: Sequences of parC alleles in fasta format.

Supplemental data 4: Number of enrollees in KPWA and number of enrollees prescribed FQ and 3GC antibiotics, for 2010-2021 by year.

Supplemental data 5: Number of Medicare enrollees in USA and in Washington state and number of beneficiaries with claims for prescribed FQ and 3GC antibiotics, for 2013-2020 by year.

Supplemental data 6: List of all samples from 2015 and 2021 study with information regarding presence of any *E. coli* or FQREC in them.

Supplemental data 7: Primers used for CH typing, gyrA-parC sequencing, PMQR, VF and BLA loci detection

Supplemental Information

Supplemental Methods

Study design and participants

Both 2015 and 2021 studies were approved by the Kaiser Permanente Washington (KPWA) Research Institute Institutional Review Board. Study protocols included waivers of consent to identify potential participants. Risks and benefits of the research were explained to participants in a mailed invite letter and consent information sheet. Participants provided informed consent for participation by sending a biological sample back in the mail. The 2015 study was carried put between July, 2015 and December, 2016, the 2021 study between May-November 2021. Initially, both studies had a cross-sectional random sample of 6,000 women ≥18 years old (2015) and 6,750 women ≥50 years old (2021), enrolled at KPWA for at least one year and neither diagnosed for UTI nor treated with antibiotics in the 12 months prior to the sample pull date in April 30, 2015 (2015 study) and April 29, 2021 (2021 study) were pre-selected. The recruitment of participants from this sample was carried out as follows: every week a random subgroup of 100-300 selectees were contacted by mail with an explanation of the project and instructions for their fecal samples to be obtained by using a self-collection kit. For 2015 study, the mailing lasted for 6 months, with 3,367 kits mailed out and 1,032 kits returned (31% response rate). Out of those, 575 kits were from 50+ yo women and thus were included in the current study. For 2021 study, 6400 kits were mailed between May and November 2021, with 1778 kits with fecal samples returned (28% response rate). The mailings were stopped early when the intended sample size of 1,000 (2015) and 1600 (2021) samples was reached.

Sample processing

Fecal samples were collected using the FecalSwab™ Sample Collection and Preservation System for Enteric Bacteria by Copan Diagnostic Inc. (Carlsbad, CA, USA). Samples were visually assessed for the quality of fecal matter before being plated on four types of agar. (1) Pre-poured HardyCHROM™ UTI agar plates (Hardy Diagnostic, USA) were used for non-antibiotic plating of *E. coli*. The proprietary composition of these plates allows for the differential detection of uropathogenic microorganisms. (2-4) For plating on ciprofloxacin, plates containing ciprofloxacin at 0.5, 2 or 10 mg/L were prepared using HiChromeTM UTI Agar (HiMedia Laboratories Pvt, Ltd., India). The rest of the sample was split into two tubes (with and without 10% glycerol) and stored at -80oC. The plates were incubated for 16-20 hours at 37°C, and up to 30 single colonies (SCs) that were morphologically identified as potential *E. coli* (magenta, pink, or opaque color) were cultured, saved, and further tested. All single colonies were tested for (a) growth on ciprofloxacin-supplemented agar and (b) clonality based on sequencing of four loci (see below). Fluoroquinolone-resistant *E. coli*

(FQREC) were defined as single isolates that grew on plates with at least 0.5 mg/L of ciprofloxacin. FQREC were further tested for ciprofloxacin MIC and susceptibility to third-generation cephalosporins (3GC) (see below). Additionally, subsets of FQREC were tested for the presence of PMQR, *bla*, and VF loci (see below).

Identification of FQREC clonality and number of QRDR mutations

E. coli clonality was determined by CH typing based on *fumC*/*fimH* sequencing [1], presence of QRDR mutations was determined by sequencing of *gyrA* and *parC* [2]. All reactions were carried out by 2-step colony PCR. Briefly, a single FQREC colony was resuspended in 50 µL of sterile water and heated at 98°C for 10 min. Primary PCR reactions were set up in a 15 µL volume using DreamTaq Mastermix (Thermofisher, USA), supplemented with 0.5 µM forward and reverse primers, and 1.5 µL of the boiled colony template. Primary PCR was run for 30 cycles under the manufacturer's recommended conditions. Subsequently, 1 µL of the PCR1 product was used for an additional PCR reaction, using nested forward and reverse primers supplemented with T7 and T7-Term tails, respectively. The nested PCR was run for 15 cycles under the same conditions, aiming to obtain a highly specific single band with T7-tailed primers suitable for downstream sequencing. The primer sequences can be found in Supplemental Table S7.

Identification of 3GCR FQREC

To identify FQREC that were non-susceptible to third-generation cephalosporins (3GCR), the isolates were tested on plates containing cefoxitin at 4 and 8 mg/L (CEF-4 and CEF-8), ceftazidime at 8 and 16 mg/L (CAZ-8 and CAZ-16), and HardyCHROM ESBL HDx (Hardy Diagnostics, USA, containing ceftazidime and cefpodoxime). The ESBL vs. non-ESBL type of 3GC-non-susceptibility was determined based on growth on the aforementioned plates and the difference in diameter growth inhibition by ceftazidime and ceftazidime/clavulanate, as described in CLSI[3].

Testing antibiotic resistance of *E. coli* isolates

Resistance of *E. coli* isolates to a panel of 12 antibiotics was tested for 383 FQREC (from 361 samples) using Kirby-Bauer disk diffusion method as described in CLSI manual[3]. The antibiotics disks were purchased from Hardy Diagnostics (Santa Maria, CA, USA) and are as follows: AMP, ampicillin, AMC, amoxicillin/clavulanate, CZ, cefazolin, IMI, imipenem, T/S, trimethoprim/sulfamethoxazole, TET, tetracycline, FOS, fosfomycin, NIT, nitrofurantoin. For a random set of 255 isolates testing was repeated.

Determining Minimum Inhibitory Concentration (MIC) of ciprofloxacin

Ciprofloxacin MIC for 390 FQREC isolates (from 369 fecal samples) was determined according to CLSI guidelines using CAMHB (BBLTM Mueller Hinton II Broth, Cation Adjusted, Becton, Dickinson and Co., MD, USA) for antibiotic concentrations 0, 0.5, 1.0,

2.0, 4.0 and 8.0 mg/L. For isolates growing at the highest 8.0 mg/L concentrations of ciprofloxacin MIC was assigned as >8.0 mg/L.

Determining presence of Plasmid-Mediated Quinolone Resistance loci (PMQR)

The PMQR (or TMQR) loci were identified using 8-loci multiplex described in Ciesielczuk et al.[4] with addition of ninth locus *qnrVC* from Kraychete et al[5]. The primers are listed in Supplemental Table 1. For testing, single colonies of FQREC isolates were resuspended in 50 µL sterile water, boiled 10 min, and 1.5 µL was used as template in a 20 µL PCR reaction with 0.5 µM primers supplied. In-house strains from the Sokurenko Lab were used as positive controls (except for *qnrVC*, for which there was no positive control). The presence of weak bands in the multiplex was confirmed or refuted in a singleplex test using appropriate primers.

Determining absence/presence of Virulence Factors (VF)

The presence of uropathogenic virulence factors (*gadA/B*[6], *iha*[7], *ireA*[8], *sat*[9], *vat*[10] and *senB*[11]) was determined by the presence of PCR products after an amplification reaction using primers listed in Supplemental Table 1. Reactions were carried out using boiled bacteria as template, as described above. In-house strains from the Sokurenko Lab were used as positive controls.

Determining absence/presence of beta-lactamase loci (BLA)

The presence of major determinants of beta-lactamases was detected using five multiplexes described in Dallenne et al.[12] Primers are listed in Supplemental Table 1. Additionally, primers specific for bla_{NDM} were added to Multiplex III. Reactions were carried out using boiled bacteria as template as described above. In-house strains from the Sokurenko Lab were used as positive controls. The presence of weak bands in multiplex was confirmed or refuted in singleplex test using appropriate primers.

Table S1. FQR *E. coli* clones identified in 2015 and 2021 studies.

Clone[a]	2015[b]	2021[b]	Clone[a]	2015[b]	2021[b]
ST131-H30	30 (38.5)	70 (20.8)	CC10-H28	0	1 (0.3)
ST1193	9 (11.5)	68 (20.2)	CC10-H29	0	1 (0.3)
ST69	7 (9)	51 (15.2)	CC10-H64	0	1 (0.3)
ST131-H41	5 (6.4)	14 (4.2)	CC10-H65	0	1 (0.3)
ST648	4 (5.1)	11 (3.3)	CC2797	0	1 (0.3)
CC10-H54	4 (5.1)	8 (2.4)	CC349	0	1 (0.3)
ST38	3 (3.8)	19 (5.7)	CC58-H0	0	1 (0.3)
CC58-H121	2 (2.6)	1 (0.3)	CC58-H1325	0	1 (0.3)
CC10-H0	1 (1.3)	5 (1.5)	CC58-H1632	0	1 (0.3)
CC10-H34	1 (1.3)	3 (0.9)	CC58-H26	0	1 (0.3)
CC21	1 (1.3)	3 (0.9)	CC58-H30	0	1 (0.3)
CC58-H61	1 (1.3)	3 (0.9)	CC58-H54	0	1 (0.3)
ST405	1 (1.3)	3 (0.9)	CH14-27	0	1 (0.3)
ST457	1 (1.3)	3 (0.9)	CH305-0	0	1 (0.3)
CC10-H23	1 (1.3)	2 (0.6)	CH40-97	0	1 (0.3)
CC10-H215	1 (1.3)	1 (0.3)	CH424-1580	0	1 (0.3)
CC58-H38	1 (1.3)	1 (0.3)	CH6-54	0	1 (0.3)
ST1163	1 (1.3)	1 (0.3)	ST101	0	1 (0.3)
CC10-H1255	1 (1.3)	0	ST117	0	1 (0.3)
CC1196	1 (1.3)	0	ST127	0	1 (0.3)
CC88	1 (1.3)	0	ST14	0	1 (0.3)
ST156	1 (1.3)	0	ST1723	0	1 (0.3)
ST95	0	7 (2.1)	ST1844	0	1 (0.3)
CC10-H27	0	6 (1.8)	ST1972	0	1 (0.3)
ST636	0	4 (1.2)	ST206	0	1 (0.3)
CC10-H30	0	3 (0.9)	ST2307	0	1 (0.3)
CC58-H32	0	2 (0.6)	ST362	0	1 (0.3)
ST2973	0	2 (0.6)	ST398	0	1 (0.3)
ST349	0	2 (0.6)	ST450	0	1 (0.3)
ST354	0	2 (0.6)	ST452	0	1 (0.3)
ST394	0	2 (0.6)	ST569	0	1 (0.3)
ST625	0	2 (0.6)	ST59	0	1 (0.3)
			ST62	0	1 (0.3)
			ST68	0	1 (0.3)
			ST73	0	1 (0.3)
			ST80	0	1 (0.3)
			ST unknown	0	1 (0.3)

[a] ST, sequence type, CC, clonal complex, H, fimH allele. *E. coli* clonality was inferred based on sequences of four loci (*fumC, fimH, gyrA, parC)* from available ~ 200K genomes in Enterobase Database (https://enterobase.warwick.ac.uk/) and ~10K sequenced isolates in Sokurenko Laboratory database (in-house data); for STs belonging to clonal complexes of ST10 (Phylogroup A) and ST58 (Phylogroup B1) a respective clonal complex is used as designation in combination with fimH allele. ‘CH’

(aka, *fumC-fimH* allele combination) is used when ST cannot be inferred based on given allele combinations; one isolate for which both *fumC* and *fimH* alleles' sequencing has failed but *gyrA* and 16S sequencing confirmed *E. coli* as species was designated as 'ST unknown'.

[b] Number of FQREC isolates with percent from total number of FQREC isolates in parenthesis is given

Table S2. Virulence loci variably present in 158 FQREC from 151 fecal samples from 2015 (N=24) and 2021 (N=127) study collections. [a]

VF combinations	No. isolates
gadA/B	20
gadA/B+iha+sat	8
gadA/B+iha+senB	3
gadA/B+sat	1
gadA/B+sat+iha+vat	1
gadA/B+senB	1
gadA/B+vat	1
iha	8
iha+gadA/B	2
iha+sat	4
iha+senB+vat	6
iha+senB	14
iha+vat	1
ireA	2
senB	22
senB+vat	6
senB+vat+ireA	1
vat	7
Total VF+	108
None found	50

[a] The tested isolates belonged to 25 clonal groups including ST131-H30 (37), ST131-H41 (10), ST1193 (16), ST69 (23), ST38 (13), CC10 (18), ST648 (10), ST95 (3), CC58 (6), ST457 (2), ST636 (2), ST349 (2), ST625 (2) and 15 other ST with single isolate belonging to them.

Table S3. PMQR determinants identified in 2015 and 2021 FQR *E. coli* isolates [a]

PMQR type	2015	2021
qnrB	27	55
qnrB + *qnrS*	1	4
qnrB + *qepA*	2	0
qnrB + *aac*6 [a]	1	2
qnrB + *oqxAB*	1	0
qnrB + *qepA* + *oqxAB*	2	0
qnrB + *qepA* + *aac*6 [a]	1	0
qnrS	7	20
qnrS + *oqxAB*	0	1
qnrD	1	0
*aac*6 [a]	3	12
qepA	0	4
oqxAB	0	1
Total	46	99

[a] *aac*6 stands for *aac(6')-Ib-cr*

Table S4. BLA loci identified in 129 3GCR FQREC isolates from 2015 and 2021. [a]

Beta-lactamase	All	H30	ST1193	ST69	Other
CTX-M-1/15	38	15	4	3	16
CTX-M-2	4	0	0	0	4
CTX-M-9/14	14	4	1	0	9
CTX-M-8/25	1	0	0	0	1
TEM	40	7	4	7	22
OXA-1	17	11	1	0	5
LAT	6	1	0	1	4
ACC-1	2	0	1	0	1
FOX	2	0	0	1	1
CIT	1	0	0	0	1
bla not found	21	3	4	1	13
bla found [b]	109	29	11	11	58

[a] BLA determinants that were tested for but not found: SHV, KPC, NDM, OXA-48, MOX

[b] Overall, in 84% of tested 3GCR isolates at least one BLA determinant was found, with 24.0% of those carrying more than one determinant; in 16% cases no genes potentially responsible for the ESBL phenotype could be identified.

Supplemental Table S5. Cumulative antibiogram of FQREC.

FQREC group	Study	No.	Resistance, % [a]							
			AMP	AMC	CZ	IMI	T/S	TET	FOS	NIT
Total	2015	78	86	40	14	0	55	47	1	0
	2021	305	77	37	**30**	1	**42**	41	3	4
H30	2015	30	77	40	17	0	37	27	0	0
	2021	64	80	44	**44**	2	31	36	2	5
ST1193	2015	9	100	22	0	0	56	56	0	0
	2021	62	63	26	8	0	32	35	2	0
Other clones	2015	39	90	44	15	0	69	62	3	0
	2021	179	81	39	**34**	2	**49**	45	3	5
≥3 QRDR mutations	2015	66	83	42	15	0	53	45	2	0
	2021	197	78	40	**32**	1	**37**	36	3	2
<3 QRDR mutations	2015	12	100	25	8	0	67	58	0	0
	2021	108	76	32	28	2	51	52	3	7

[a] FQREC isolates were tested to for full or intermediate level of resistance to antibiotics using disk diffusion method, and the overall or group-specific non-susceptibility was calculated as percent of non-susceptible isolates from total tested; AMP, ampicillin, AMC, amoxicillin/clavulanate, CZ, cefazolin, IMI, imipenem, T/S, trimethoprim/sulfamethoxazole, TET, tetracycline, FOS, fosfomycin, NIT, nitrofurantoin. Changes in resistance level between 2015 and 2021 FQREC which are statistically significant (P<.05) are shown in bold red (increase in resistance) or green color (decrease in resistance).

Table S6. Types of QRDR mutations and their prevalence across major FQREC clonal groups.

Category		Total	Genotype 1 (No.)	Genotype 2 (No.)	Genotype 3 (No.)	Genotype 4 (No.)	Genotype 5 (No.)
**ST131-*H*30**							
	**≥3 QRDR**	**99**	GyrA:S83L,D87N, ParC:S80I,E84V (n=99)				
	**1 QRDR**	**1**	GyrA:S83L (n=1)				
**ST1193**		**77**	GyrA:S83L,D87N, ParC:S80I (n=77)				
**ST69**							
	**Total**	**58**					
	**≥3 QRDR**	**12**	GyrA:S83L,D87N, ParC:S80I (n=9)	GyrA:S83L,D87Y, ParC:S80I (n=2)	GyrA:S83L,D87G, ParC:S80I (n=1)		
	**2 QRDR**	**32**	GyrA:S83L, ParC:S80I (n=17)	GyrA:S83L, ParC:E84G (n=15)			
	**1 QRDR**	**4**	GyrA:S83L (n=4)				
	**0 QRDR**	**10**	no QRDR (n=10)				
**Other**							
	**Total**	**176**					
	**≥3 QRDR**	**91**	GyrA:S83L,D87N, ParC:S80I (n=70)	GyrA:S83L,D87Y, ParC:S80I (n=12)	GyrA:S83L,D87H, ParC:S80I (n=4)	GyrA:S83L,D87N, ParC:S80I,E84G (n=4)	GyrA:S83L,D87N, ParC:S80I,E84K (n=1)
	**2 QRDR**	**6**	GyrA:S83L, ParC:S80I (n=5)	GyrA:S83V, ParC:S80I (n=1)			
	**1 QRDR**	**53**	GyrA:S83L (n=48)	GyrA:S83A (n=2)	GyrA:S83I (n=2)	GyrA:S83V (n=1)	
	**0 QRDR**	**26**	no QRDR (n=26)				

SUPPLEMENTARY REFERENCES

1. Weissman, S.J., *et al.* High-resolution two-locus clonal typing of extraintestinal pathogenic *Escherichia coli*. *Appl Environ Microbiol* **78**, 1353-1360 (2012).
2. Johnson, J.R., *et al.* Abrupt emergence of a single dominant multidrug-resistant strain of *Escherichia coli*. *J Infect Dis* **207**, 919-928 (2013).
3. CLSI. Performance standards for antimicrobial susceptibility testing; twentieth information supplement. Vol. 30 (2010).
4. Ciesielczuk, H., Hornsey, M., Choi, V., Woodford, N. & Wareham, D.W. Development and evaluation of a multiplex PCR for eight plasmid-mediated quinolone-resistance determinants. *J Med Microbiol* **62**, 1823-1827 (2013).
5. Kraychete, G.B., Botelho, L.A., Campana, E.H., Picao, R.C. & Bonelli, R.R. Updated Multiplex PCR for Detection of All Six Plasmid-Mediated qnr Gene Families. *Antimicrob Agents Chemother* **60**, 7524-7526 (2016).
6. Grant, M.A., Weagant, S.D. & Feng, P. Glutamate decarboxylase genes as a prescreening marker for detection of pathogenic Escherichia coli groups. *Appl Environ Microbiol* **67**, 3110-3114 (2001).
7. Johnson, J.R., *et al.* Molecular epidemiological and phylogenetic associations of two novel putative virulence genes, iha and iroN(E. coli), among Escherichia coli isolates from patients with urosepsis. *Infect Immun* **68**, 3040-3047 (2000).
8. Russo, T.A., Carlino, U.B. & Johnson, J.R. Identification of a new iron-regulated virulence gene, ireA, in an extraintestinal pathogenic isolate of Escherichia coli. *Infect Immun* **69**, 6209-6216 (2001).
9. Tanih, N.F., *et al.* Prevalence of Virulence Genes in Enteroaggregative Escherichia coli Isolates from Young Children from Rural South Africa. *Am J Trop Med Hyg* **101**, 1027-1033 (2019).
10. Paixao, A.C., *et al.* Detection of virulence-associated genes in pathogenic and commensal avian Escherichia coli isolates. *Poult Sci* **95**, 1646-1652 (2016).
11. Cusumano, C.K., Hung, C.S., Chen, S.L. & Hultgren, S.J. Virulence plasmid harbored by uropathogenic Escherichia coli functions in acute stages of pathogenesis. *Infect Immun* **78**, 1457-1467 (2010).
12. Dallenne, C., Da Costa, A., Decre, D., Favier, C. & Arlet, G. Development of a set of multiplex PCR assays for the detection of genes encoding important beta-lactamases in Enterobacteriaceae. *J Antimicrob Chemother* **65**, 490-495 (2010).

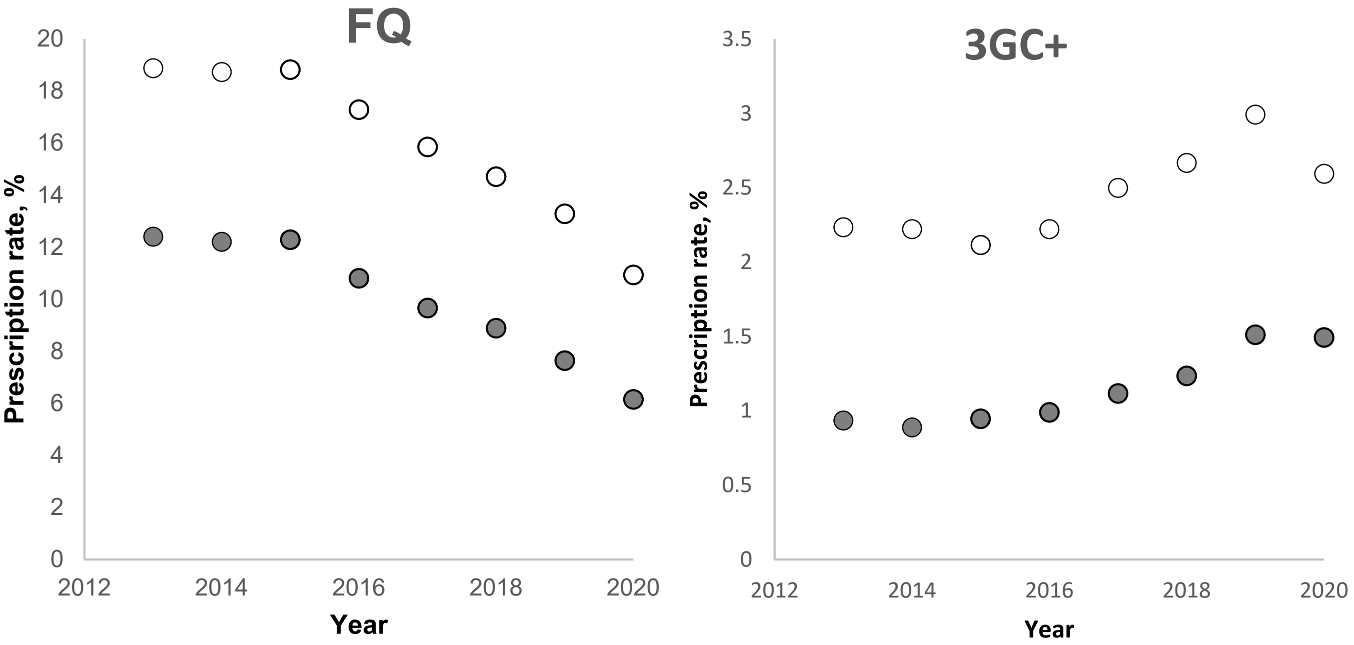

Supplemental Figure S1. Prescription rates of fluoroquinolones (FQ) and third and higher generation cephalosporins (including carbapenems, 3GC+) among Medicare enrollees in 2013-2020 in Washington state (closed circles) and USA (open circles).

The rate is calculated as percent of total beneficiaries (the total number of unique Medicare Part D beneficiaries with at least one claim for the drug per year) from total number of enrollees per year. All data were downloaded from http://data.cms.gov:

- The CMS Program Statistics - Medicare Part D tables provide use and Part D drug costs by type of Part D plan (stand-alone prescription drug plan and Medicare
- The CMS Program Statistics - Medicare Part D Enrollment tables provide data on characteristics of the Medicare Part D covered population.
