# Supplementary material for: Increase in the community circulation of ciprofloxacin-resistant Escherichia coli despite reduction in antibiotic prescriptions
Source: Commun Med (Lond). 2023 Aug 12;3:110. doi: 10.1038/s43856-023-00337-2 (PMC10421857; doi:10.1038/s43856-023-00337-2)
Supplement: Supplementary file 10 — Description of Additional Supplementary Files [file 43856_2023_337_MOESM10_ESM.pdf]

Description of Additional Supplementary Files

Supplemental Data 1: List of FQREC from 2015 and 2021 fecal samples with the results of any testing performed on them (sequencing, antimicrobial resistance, PMQR, BLA and VF determinants, number and type of QRDR mutations in GyrA and ParC and ciprofloxacin MIC.

Supplemental data 2: Sequences of gyrA alleles in fasta format**.**

Supplemental data 3: Sequences of parC alleles in fasta format.

Supplemental data 4: Number of enrollees in KPWA and number of enrollees prescribed FQ and 3GC antibiotics, for 2010-2021 by year.

Supplemental data 5: Number of Medicare enrollees in USA and in Washington state and number of beneficiaries with claims for prescribed FQ and 3GC antibiotics, for 2013-2020 by year.

Supplemental data 6: List of all samples from 2015 and 2021 study with information regarding presence of any *E. coli* or FQREC in them.

Supplemental data 7: Primers used for CH typing, gyrA-parC sequencing, PMQR, VF and BLA loci detection
