# Supplementary material for: Increase in the community circulation of ciprofloxacin-resistant Escherichia coli despite reduction in antibiotic prescriptions
Source: Commun Med (Lond). 2023 Aug 12;3:110. doi: 10.1038/s43856-023-00337-2 (PMC10421857; doi:10.1038/s43856-023-00337-2)
Supplement: Supplementary file 11 — Reporting Summary [file 43856_2023_337_MOESM11_ESM.pdf]

nature portfolio

Corresponding author(s): *Double-anonymous peer review submissions: write DAPR and your manuscript number here instead of author names.*

Last updated by author(s): *YYYY-MM-DD*

Reporting Summary

Nature Portfolio wishes to improve the reproducibility of the work that we publish. This form provides structure for consistency and transparency in reporting. For further information on Nature Portfolio policies, see our Editorial Policies and the Editorial Policy Checklist.

Statistics

For all statistical analyses, confirm that the following items are present in the figure legend, table legend, main text, or Methods section.

n/a	Confirmed	
☐	☒	The exact sample size (*n*) for each experimental group/condition, given as a discrete number and unit of measurement
☒	☐	A statement on whether measurements were taken from distinct samples or whether the same sample was measured repeatedly
☐	☒	The statistical test(s) used AND whether they are one- or two-sided<br>*Only common tests should be described solely by name; describe more complex techniques in the Methods section.*
☐	☒	A description of all covariates tested
☒	☐	A description of any assumptions or corrections, such as tests of normality and adjustment for multiple comparisons
☐	☒	A full description of the statistical parameters including central tendency (e.g. means) or other basic estimates (e.g. regression coefficient) AND variation (e.g. standard deviation) or associated estimates of uncertainty (e.g. confidence intervals)
☐	☒	For null hypothesis testing, the test statistic (e.g. *F*, *t*, *r*) with confidence intervals, effect sizes, degrees of freedom and *P* value noted<br>*Give P values as exact values whenever suitable.*
☒	☐	For Bayesian analysis, information on the choice of priors and Markov chain Monte Carlo settings
☒	☐	For hierarchical and complex designs, identification of the appropriate level for tests and full reporting of outcomes
☒	☐	Estimates of effect sizes (e.g. Cohen's *d*, Pearson's *r*), indicating how they were calculated

Our web collection on statistics for biologists contains articles on many of the points above.

Software and code

Policy information about availability of computer code

Data collection	SAS software was used to create scripts for (1) data pull of antibiotic prescriptions in KPWA, (2) KPWA enrollees eligible for the recruitment into the study
Data analysis	STATA software v14.2 (StataCorp LP, TX, USA) was used for statistical analysis; PowerBI (Microsoft, WA, USA) was used to analyze prescription data.

For manuscripts utilizing custom algorithms or software that are central to the research but not yet described in published literature, software must be made available to editors and reviewers. We strongly encourage code deposition in a community repository (e.g. GitHub). See the Nature Portfolio guidelines for submitting code & software for further information.

Data

Policy information about availability of data

All manuscripts must include a data availability statement. This statement should provide the following information, where applicable:

- Accession codes, unique identifiers, or web links for publicly available datasets
- A description of any restrictions on data availability
- For clinical datasets or third party data, please ensure that the statement adheres to our policy

The source data on both studies underlying the tables and graphs are supplied as a Supplementary Data 1-7 files

Research involving human participants, their data, or biological material

Policy information about studies with human participants or human data. See also policy information about sex, gender (identity/presentation), and sexual orientation and race, ethnicity and racism.

Reporting on sex and gender	Present study includes only female participants of 50+ year of age. No sex- or gender-based analysis was either performed or reported.
Reporting on race, ethnicity, or other socially relevant groupings	N/A
Population characteristics	Besides female sex and age above 50, other recruiting parameters for the study were (a) absence of any documented antibiotic prescription within last 12 months, (b) not residing in hospice care, and (c) have documented agreement to be contacted for participation in a research study.
Recruitment	Eligible candidates were mailed a package containing the brochure which describes the study and its importance, a self-administered collection kit for fecal sample, and a return envelope. Candidates were explained that by returning their kit the were consenting to participate in the study.
Ethics oversight	This study was approved by the Kaiser Permanente Washington (KPWA) Research Institute Institutional Review Board. Candidates were explained that by returning their kit the were consenting to participate in the study.

Note that full information on the approval of the study protocol must also be provided in the manuscript.

Field-specific reporting

Please select the one below that is the best fit for your research. If you are not sure, read the appropriate sections before making your selection.

☒ Life sciences ☐ Behavioural & social sciences ☐ Ecological, evolutionary & environmental sciences

For a reference copy of the document with all sections, see nature.com/documents/nr-reporting-summary-flat.pdf

Life sciences study design

All studies must disclose on these points even when the disclosure is negative.

Sample size	The sample size was calculated based on previously determined rates: 6,400 potential participants identified to receive and process 1,600 fecal samples (based on average 25% response rate) to identify around 150 of pandemic drug-resistant clones (based on average 9% carriage rate).
Data exclusions	No data were excluded from analysis
Replication	No reproducibility analysis is required with the study design
Randomization	There was no allocation of participants into different experimental groups
Blinding	Blinding was not required since there was no allocation into different groups

Reporting for specific materials, systems and methods

We require information from authors about some types of materials, experimental systems and methods used in many studies. Here, indicate whether each material, system or method listed is relevant to your study. If you are not sure if a list item applies to your research, read the appropriate section before selecting a response.

Materials & experimental systems

n/a	Involved in the study	
☒	☐	Antibodies
☒	☐	Eukaryotic cell lines
☒	☐	Palaeontology and archaeology
☒	☐	Animals and other organisms
☒	☐	Clinical data
☒	☐	Dual use research of concern
☒	☐	Plants

Methods

n/a	Involved in the study	
☒	☐	ChIP-seq
☒	☐	Flow cytometry
☒	☐	MRI-based neuroimaging
